# Supplementary material for: Clinical and molecular characteristics of COVID-19 patients with persistent SARS-CoV-2 infection
Source: Nat Commun. 2021 Jun 9;12:3501. doi: 10.1038/s41467-021-23621-y (PMC8190301; doi:10.1038/s41467-021-23621-y)
Supplement: Supplementary file 1 — Supplementary Information [file 41467_2021_23621_MOESM1_ESM.pdf]

Clinical and molecular characteristics of COVID-19 patients with persistent SARS-CoV-2 infection

Authors and Affiliations

Bin Yang[1,2], Junpeng Fan[1,2], Jia Huang[1,2], Ensong Guo[1,2], Yu Fu[1,2], Si Liu[1,2], Rourou Xiao[1,2], Chen Liu[1,2], Funian Lu[1,2], Tianyu Qin[1,2], Chao He[1,2], Zizhuo Wang[1,2], Xu Qin [1,2], Dianxing Hu [1,2], Lixin You[1,2], Xi Li[1,2], Tian Wang[1,2], Peng Wu[1,2], Gang Chen[1,2], Jianfeng Zhou[3], Kezhen Li[1,2*], Chaoyang Sun[1,2*]

1. Department of Obstetrics and Gynecology, Tongji Hospital, Tongji Medical College, Huazhong University of Science and Technology, Wuhan 430030, China.
2. Cancer Biology Research Center, Tongji Hospital, Tongji Medical College, Huazhong University of Science and Technology, Wuhan 430030, China
3. Department of Hematology, Tongji Hospital, Tongji Medical College, Huazhong University of Science and Technology, Wuhan 430030, China

These authors contribute equally: Bin Yang, Junpeng Fan, and Jia Huang

These authors jointly supervised this work: Kezhen Li, Chaoyang Sun. Correspondence should be addressed to K.L. (email: tjkeke@126.com) or to C.S. (email: suncydoctor@gmail.com).

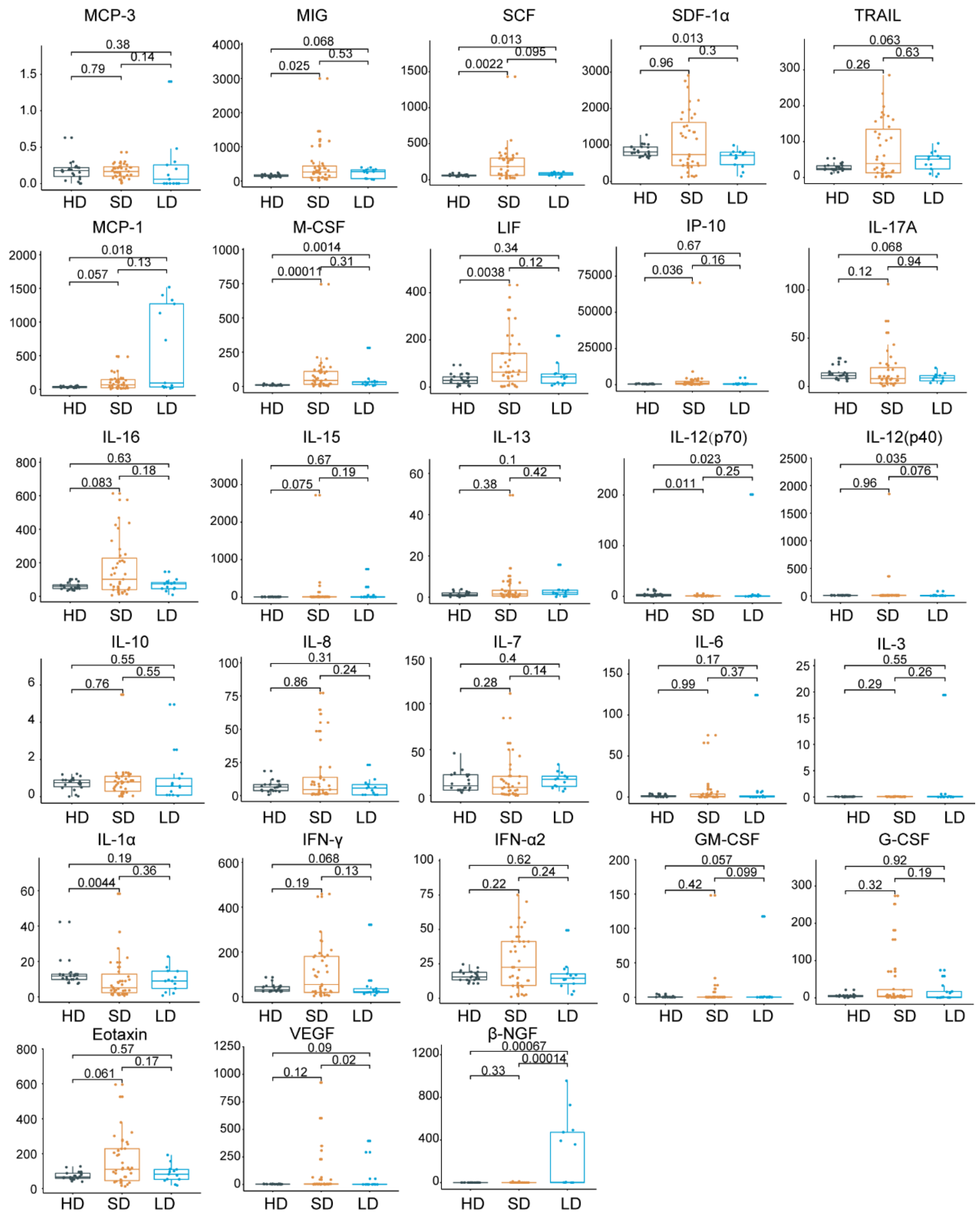

Supplementary Fig. 1 Clinical and cytokines/chemokines characteristics in LDs and SDs.

Samples from HDs (n=22) LDs (n=12) and SDs (n=38) were collected, and assays were performed to measure the concentrations of 48 cytokines/chemokines. The box plots show the median (middle line) and the first and third quartiles (boxes), whereas the whiskers show 1.5× the IQR above and below the box. One-side Wilcoxon rank-sum test are performed.

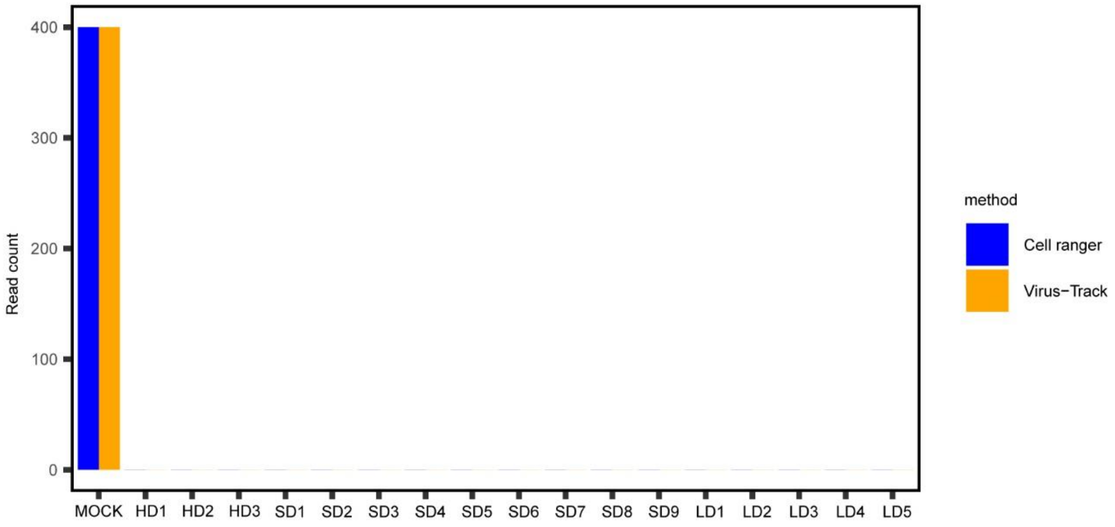

Supplementary Fig. 2 SARS-CoV-2 transcripts detected in samples using Virus-Track and Cell ranger. Mock sample with SARS-CoV-2 transcripts was generated by add 200 SARS-CoV-2 paired reads to the health control. SARS-CoV-2 transcripts were identified from sequencing data using Viral-Track (yellow) and Cell Ranger (blue) with a modified reference contain SARS-CoV-2 genome (NC_045512.2).

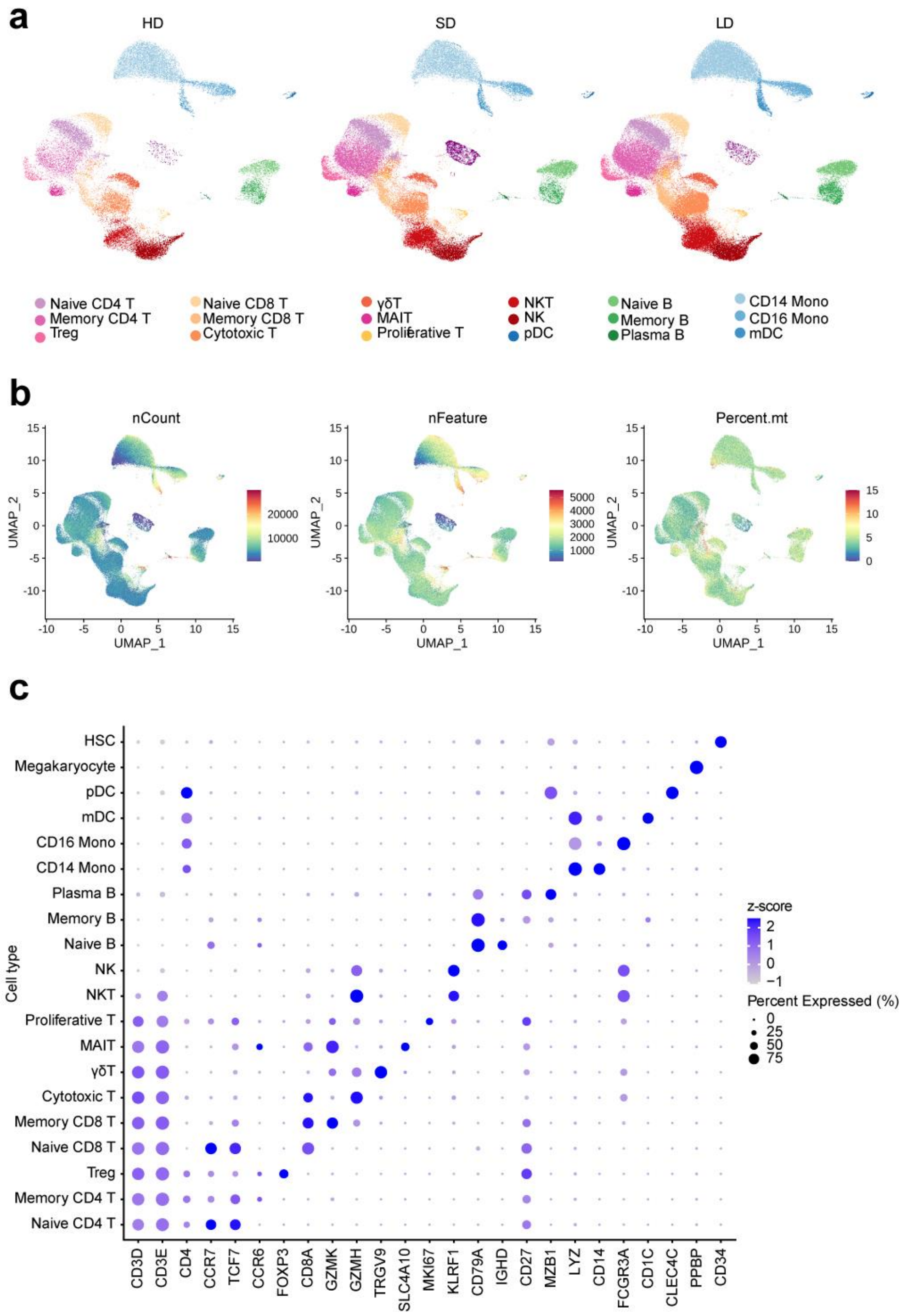

Supplementary Fig.3 Quality of clustering and canonical markers expression in PBMCs for healthy donors and COVID-19 patients a, UMAP plot of the HDs, SDs, LDs conditions. Each dot corresponds to a single cell, colored by cell type. **b**, UMAP plot of UMIs gene counts and percentage of mitochondrial genes in all cells. **c**, Dot plot of canonical cell markers. The color intensity indicates the expression level of each marker and the point size indicates the ratio of cells that has marker expressed.

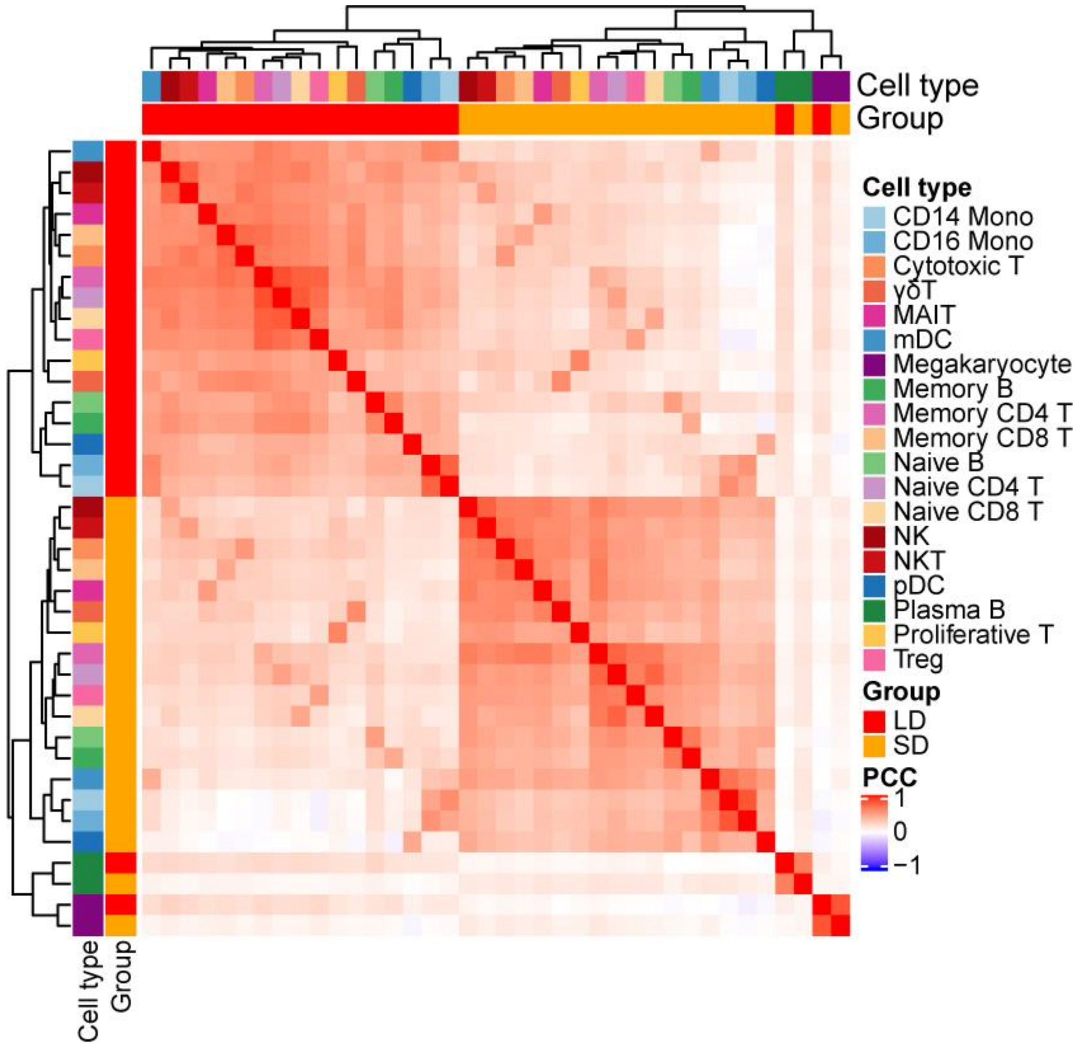

Supplementary Fig.4 Differences in cell compositions by Single-cell transcriptomes of PBMCs. Hierarchical clustering using the PCC of a normalized transcriptome between disease groups at cell type resolution. The color intensity indicates the PCC and the color bars above the heatmap indicate the cell type and disease group.

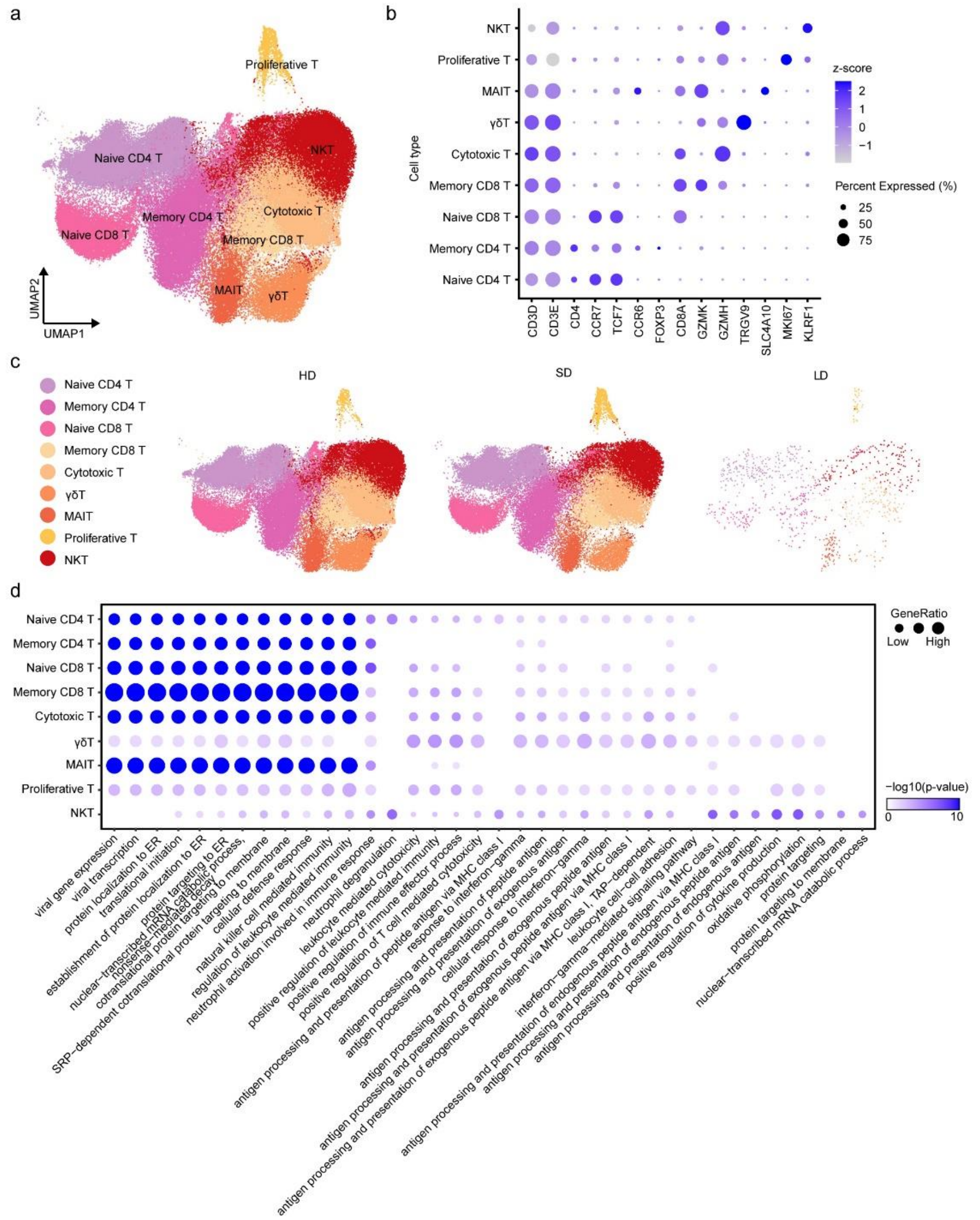

Supplementary Fig.5 Transcriptional signatures associated with long viral shedding duration of published data. a, UMAP plot of single cells colored by cell types identified from HDs (n=20), SDs (n=16), LDs (n=2). **b**, Dot plot of canonical cell markers. The color intensity indicates the expression level of each marker and the point size indicates the ratio of cells that has marker expressed. **c**, UMAP plot of the HDs, SDs, LDs conditions. Each dot corresponds to a single cell, colored by cell type. **d**, Enriched GO pathways of down-regulated genes between LDs and HDs at cell type resolution. Pathways enriched by ribosomal genes are labeled in red. The color intensity indicates the enrichment *p*-values and the point size indicates the ratio of gene enrich in each pathway. **e**, Expression level of selected genes across 7 samples. The color intensity indicates the relative expression level.

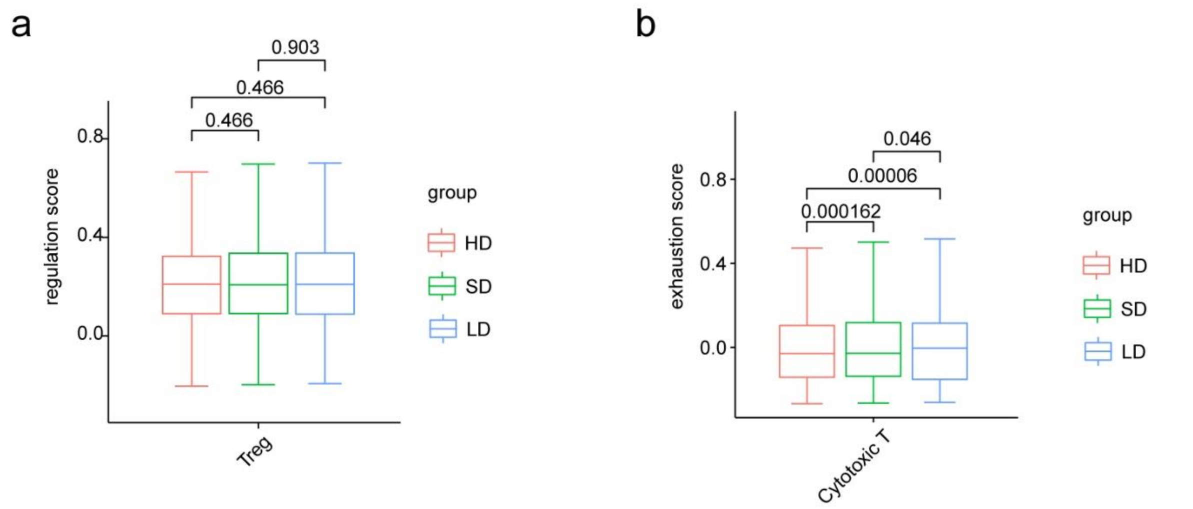

Supplementary Fig.6 Compare regulation and exhaustion score in each group. a. Box plot of regulation scores for each group (HDs (n=3), SDs (n=9), LDs (n=5)) in Treg. **b.** Box plot of exhaustion scores for each group (HDs (n=3), SDs (n=9), LDs (n=5)) in cytotoxic T cells. The box plots show the median (middle line) and the first and third quartiles (boxes), whereas the whiskers show 1.5× the IQR above and below the box. One-side Wilcoxon rank-sum test are performed.

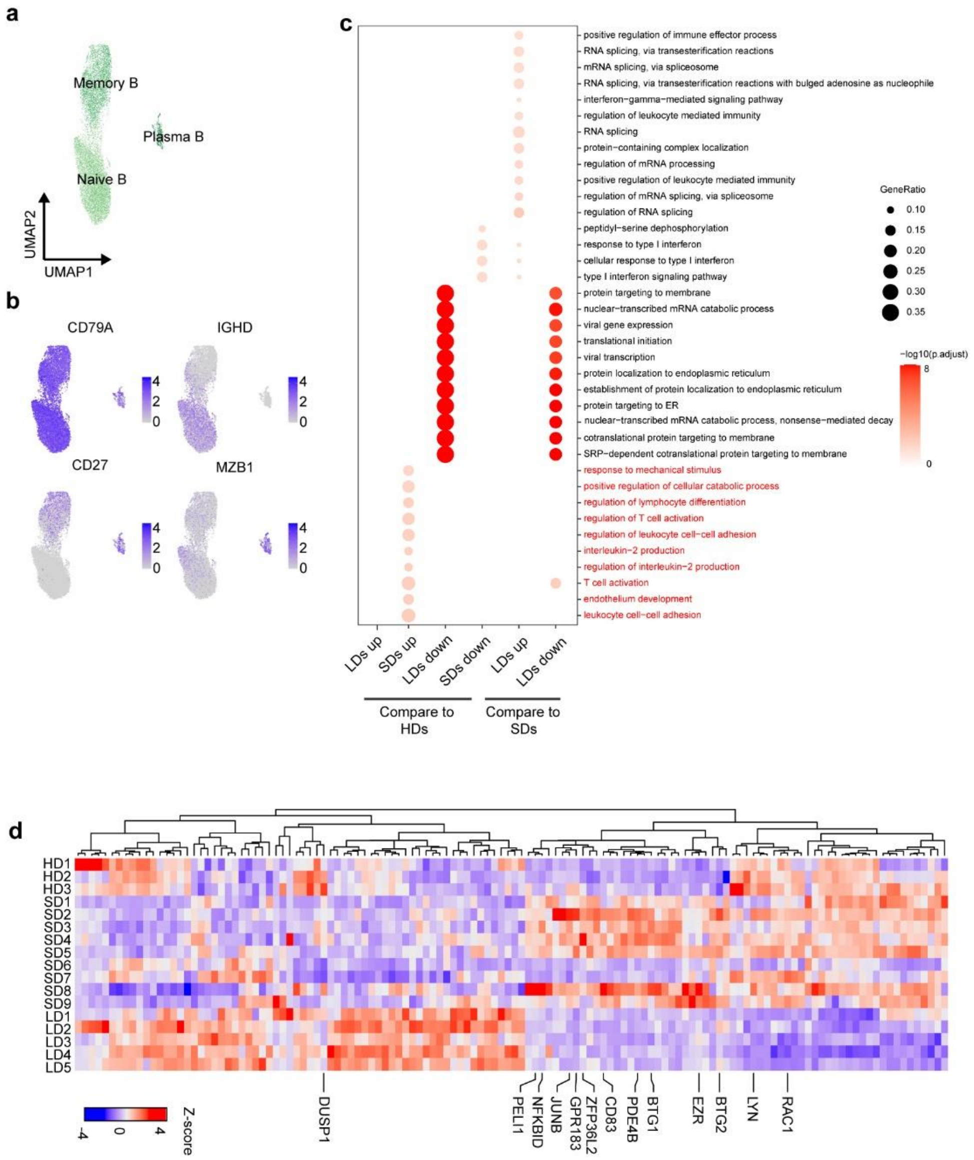

Supplementary Fig.7 Immunological features of B cell subsets. a, UMAP projection of B cells. Each dot corresponds to a single cell, colored by cell type. **b**, UMAP plot of canonical markers in 3 cell clusters. Data are colored according to log scaled expression levels. **c**, Enriched GO pathways of COVID-19 groups in memory B cells (top 2 columns: DEGs between LDs and SDs, bottom 4 columns: DEGs for SDs and LDs compared to HDs. Pathway enriched by SDs up-regulated genes are labeled in red. The color intensity indicates the enrichment *p*-values and the point size indicates the ratio of gene enrich in each pathway. **d**, Hierarchical clustering of expression of DEGs in memory B cells at sample level. The color intensity indicates the relative expression of each gene.

Supplementary Table 1 Demographic characteristics and laboratory findings of enrolled patients with cytokine measurements.

		Normal range	SDs (n=38)	LDs (n=12)	*p* value#
Age (years, median, (IQR))			57(38.25-67.75)	59.5(54-63.25)	0.33
Sex*					0.73
	Female		20	7	
	Male		18	5	
Comorbidities*					
	Hypertension,%		10(26.3)	6(50)	0.12
	Cardiovascular disease,%		5(13.2)	1(8.3)	0.95
	Diabetes,%		7(18.4)	5(41.7)	0.1
Durantion of viral shedding (days,median,IQR)			16(11.25-19)	57(53-76.5)	0
Blood routine	Whiteblood count, x 10^9/L	3.5-9.5	6.17±2.52	5.86±2.58	0.71
	Neutrophil count, x 10^9/L	1.8-6.3	4.36±2.51	4.17±2.48	0.81
	Lymphocyte count, x 10^9/L	1.1-3.2	1.22±0.55	1.19±0.50	0.85
	Platelet count, x 10^9/L	125-350	210.65±84.5	188.8±87.9	0.45
	Hemoglobin, g/L	115-150	128.9±28.1	120.7±19.3	0.35
Coagulation function	Prothrombin time,s	11.5-14.5	13.78±0.78	13.63±0.71	0.062
	Activated partial thromboplastin time, s	29-42	42.23±5.18	38.61±4.36	0.055
	D-dimer, mg/L	< 0.5	1.12±1.86	1.63±3.17	0.527
Blood biochemistry	Alanine aminotransferase, U/L	≤33	31.39±32.6	33±28	0.88
	Aspartate aminotransferase, U/L	≤32	26±16.64	22.42±6.69	0.48
	Lactate dehydrogenase, U/L	135-214	236.8±54.23	242.08±145.25	0.86
Infection-related biomarkers	Procalcitonin,ng/ml	< 0.05	0.55±1.73	1.05±2.41	0.51
	CRP,mg/L	≤10	35.02±48.32	55.02±64.88	0.26
	Erythrocyte sedimentation rate,mm/H	0-20	32.27±28	45.4±33.89	0.22

IQR： the interquartile range; CRP: C-reactive protein. **p* values obtained by Fisher's exact test and the rest of #*p* values obtained by two-tailed unpaired student-t test.

Supplemental Table 2 Demographic characteristics of people who have been performed single-cell sequencing

	Normal Range	Long-term infection					Short-term infection									Healthy donor		
		LD1	LD2	LD3	LD4	LD5	SD1	SD2	SD3	SD4	SD5	SD6	SD7	SD8	SD9	HD1	HD2	HD3
**Sequenced cell number**		11886	11029	10352	13534	9196	8419	8462	11153	8407	7952	10453	8905	9523	8137	9628	10345	10565
**Characteristics**																		
Age(years)		64	54	58	36	55	57	59	74	59	56	41	26	65	60	36	43	29
Gender		M	F	F	F	F	F	F	F	M	F	F	F	F	F	F	F	F
Symptoms onset		Fever, cough	Cough, fatigue	Fever,cough, Chills	Sore throat, cough, fatigue	Fever, fatigue, cough, short of breath	Fever	Fever, cough, chills, fatigue	Fever	Fever, Diarrhea	Cough	Cough	Fever, Cough	Fever, cough	fever, emesis, stomachache, fatigue	NA	NA	NA
Severity		general	general	general	severe	critical	severe	general	critical	general	critical	mild	severe	critical	severe	NA	NA	NA
Durantion of viral shedding, days		88	100	54	76	77	2	4	22	3	5	9	17	8	18	NA	NA	NA
Onset of symptom to sampling, days		90	102	56	78	80	98	89	70	85	89	71	62	95	55	NA	NA	NA
**Blood routine**																		
leukocyte, $\times 10^9$/L	3.50-9.50	6.31	6.11	4.81	4.21	5.41	7.78	5.09	3.62	5.95	19.91	7.59	2.85	4.81	3.46	NA	NA	NA
neutrophil,%	40.0-75.0	55.6	57.6	64.6	61.5	63.3	58	54.5	62.7	58.1	91.4	72.5	59.9	64.6	50.6	NA	NA	NA
neutrophil,$\times 10^9$/L	1.80-6.30	3.51	3.52	3.11	2.59	3.42	4.51	2.77	2.77	3.46	18.21	5.5	1.71	3.11	1.75	NA	NA	NA
lymphocyte,%	20.0-50.0	31.5	33.4	28.9	30.6	28.8	29.3	32.4	21	22.2	2.8	19.2	23.9	28.9	29.2	NA	NA	NA

lymphocyte,× 10^9/L	1.10-3.20	1.99	2.04	1.39	1.29	1.56	2.28	1.65	0.76	1.32	0.55	1.46	0.68	1.39	1.01	NA	NA	NA
monocyte,%	3.0-10.0	9.5	7.22	4.2	5	5.5	7.6	8.6	11.9	11.8	5.1	7.5	14	4.2	15	NA	NA	NA
monocyte,× 10^9/L	0.10-0.60	0.6	0.44	0.2	0.21	0.3	0.59	0.44	0.43	0.7	1.01	0.57	0.4	0.2	0.52	NA	NA	NA
eosinophil,%	0.4-8.0	2.9	1.3	1.9	2.4	2	4.5	4.1	4.1	7.7	0.6	0.7	1.8	1.9	4.9	NA	NA	NA
eosinophil,× 10^9/L	0.02-0.52	0.18	0.08	0.09	0.1	0.11	0.35	0.21	0.15	0.46	0.12	0.05	0.05	0.09	0.17	NA	NA	NA
basophilic granulocyte,%	0.0-1.0	0.5	0.5	0.4	0.5	0.4	0.6	0.4	0.3	0.2	0.1	0.1	0.4	0.4	0.3	NA	NA	NA
basophilic granulocyte,× 10^9/L	0.00-0.10	0.03	0.03	0.02	0.02	0.02	0.05	0.02	0.01	0.01	0.02	0.01	0.01	0.02	0.01	NA	NA	NA
PLT, × 10^9/L	125.0-350.0	139	235	137	208	214	230	179	224	278	209	343	176	137	175	NA	NA	NA
**Coagulation function**																		
Prothrombin time, s	11.5-14.5	15.6	14.4	NA	13.9	13.5	13.2	13.8	12.9	13.2	15.3	NA	12.7	NA	13.9	NA	NA	NA
Prothrombin Time and International Normalized Ratio (PT/INR)	0.80-1.20	1.23	1.12	NA	1.06	1.03	0.99	1.05	0.96	1	1.22	NA	0.96	NA	1.06	NA	NA	NA
Fibrinogen, g/L	2.00-4.00	2.69	3.39	NA	2.27	3.38	3.67	2.96	3.16	3.13	8.92	NA	4.96	NA	2.27	NA	NA	NA
Activated partial thromboplastin, s	29-42	39.6	37.9	NA	39	34.8	38.4	40.6	45.4	40.4	43.1	NA	39.3	NA	39	NA	NA	NA
Thrombin time, s	14-19	15.9	16.7	NA	14.9	14.7	16.5	16.8	14.6	14.6	15.9	NA	14.9	NA	14.9	NA	NA	NA
D-dimer, μg/ml	<0.5	<0.22	0.53	NA	<0.22	0.69	0.32	<0.22	0.54	0.25	> 21	NA	0.62	NA	<0.22	NA	NA	NA

M: male; F: female; NA: not available; PLT: Platelet Count Test.
