# Supplementary material for: Clinical and molecular characteristics of COVID-19 patients with persistent SARS-CoV-2 infection
Source: Nat Commun. 2021 Jun 9;12:3501. doi: 10.1038/s41467-021-23621-y (PMC8190301; doi:10.1038/s41467-021-23621-y)
Supplement: Supplementary file 3 — Reporting Summary [file 41467_2021_23621_MOESM3_ESM.pdf]

nature research

Corresponding author(s): Chaoyang Sun

Last updated by author(s): Apr 13, 2021

# Reporting Summary

Nature Research wishes to improve the reproducibility of the work that we publish. This form provides structure for consistency and transparency in reporting. For further information on Nature Research policies, see our Editorial Policies and the Editorial Policy Checklist.

## Statistics

For all statistical analyses, confirm that the following items are present in the figure legend, table legend, main text, or Methods section.

| n/a | Confirmed | |
|---|---|---|
| [ ] | [x] | The exact sample size (*n*) for each experimental group/condition, given as a discrete number and unit of measurement |
| [ ] | [x] | A statement on whether measurements were taken from distinct samples or whether the same sample was measured repeatedly |
| [ ] | [x] | The statistical test(s) used AND whether they are one- or two-sided<br>*Only common tests should be described solely by name; describe more complex techniques in the Methods section.* |
| [ ] | [x] | A description of all covariates tested |
| [ ] | [x] | A description of any assumptions or corrections, such as tests of normality and adjustment for multiple comparisons |
| [ ] | [x] | A full description of the statistical parameters including central tendency (e.g. means) or other basic estimates (e.g. regression coefficient) AND variation (e.g. standard deviation) or associated estimates of uncertainty (e.g. confidence intervals) |
| [ ] | [x] | For null hypothesis testing, the test statistic (e.g. $F$, $t$, $r$) with confidence intervals, effect sizes, degrees of freedom and $P$ value noted<br>*Give P values as exact values whenever suitable.* |
| [x] | [ ] | For Bayesian analysis, information on the choice of priors and Markov chain Monte Carlo settings |
| [ ] | [x] | For hierarchical and complex designs, identification of the appropriate level for tests and full reporting of outcomes |
| [ ] | [x] | Estimates of effect sizes (e.g. Cohen's $d$, Pearson's $r$), indicating how they were calculated |

*Our web collection on statistics for biologists contains articles on many of the points above.*

## Software and code

Policy information about availability of computer code

**Data collection**

PBMCs isolated from 14 patients and 3 healthy donors as controls were performed 10x scRNA-seq. Chromium Single-Cell V(D)J Enrichment kit according to the manufacturer's protocol (10x Genomics).The final libraries were sequenced using the MGISEQ-2000RS platform (BGIShenzhen, China)

**Data analysis**

1:SARS-CoV-2 transcripts were identified from sequencing data using Viral-Track and Cell Ranger (version 3.0.1, 10x Genomics) with a modified reference contain SARS-CoV-2 genome (NC_045512.2)
2:Quality of cells were then assessed based on the UMI counts per cell, genes expressed per cell and the proportion of mitochondrial gene counts using Seurat (version 4.0.0)
3:the single-cell sequencing dataset was integrated using Harmony (version 1.0)
4:DEGs were performed using "FindMarkers" function with MAST algorithm in Seurat (version 4.0.0)
5:The TCR (VJ) sequencing data were performed using Cell Ranger V(D)J pipeline with GRCh38 as reference
6:One-side Wilcoxon rank-sum test were performed using R (version 4.0.2) in this study
7:The two-sided log-rank test and Non-paired two-tailed student-t test were performed using GraphPad Prism (version 8.0.2) in Figure 1a/b.
8:Non-paired two-tailed student-t test and Fisher's exact test was performed using SPSS (version 22.0) in supplementary table 1.
9:Bio-Plex Manager software (version 6.2);
10:clusterProfiler software (version 3.1.8.1)
11:Computer code is available from GitHub under https://github.com/FlyPythons/SingleCell_COVID19_paper_scripts.

For manuscripts utilizing custom algorithms or software that are central to the research but not yet described in published literature, software must be made available to editors and reviewers. We strongly encourage code deposition in a community repository (e.g. GitHub). See the Nature Research guidelines for submitting code & software for further information.

## Data

Policy information about availability of data

All manuscripts must include a data availability statement. This statement should provide the following information, where applicable:

- Accession codes, unique identifiers, or web links for publicly available datasets
- A list of figures that have associated raw data
- A description of any restrictions on data availability

Data Availability
All data are available within the Article and Supplementary Files which include an additional Excel file containing Source Data and all raw data. The single-cell sequencing RNA data have been deposited to the European Bioinformatics Institute (EMBL-EBI): PRJEB44229 ERP128255. We have also downloaded and analysis scRNA-seq data from NCBI GEO database (GSE158055) and bulk RNA-seq data of 103 COVID-19 patients from EMBL-EBI: ERP127339. Source data are provided with this paper.

# Field-specific reporting

Please select the one below that is the best fit for your research. If you are not sure, read the appropriate sections before making your selection.

☒ Life sciences ☐ Behavioural & social sciences ☐ Ecological, evolutionary & environmental sciences

For a reference copy of the document with all sections, see nature.com/documents/nr-reporting-summary-flat.pdf

# Life sciences study design

All studies must disclose on these points even when the disclosure is negative.

| | |
|---|---|
| Sample size | 1: Cytokine measurements: As of April 30, 2020, serum from 50 patients who signed an informed consent form during hospitalization. A total of 12 non-critical COVID-19 in-patients exhibited long duration of viral shedding (LDs, >45 days). We also collected 38 age- and gender-matched non-critical COVID-19 in-patients whose viral shedding durations (SDs) were less than 21 days for comparison. All the patients were identified as laboratory-confirmed and signed an informed consent form at Tongji Hospital, Wuhan, China. And 22 healthy donors who signed an informed consent form as controls.<br>2:scRNA-seq: PBMCs isolated from 14 patients and 3 healthy donors as controls who signed an informed consent form were performed 10X scRNA-seq. A total of 5 COVID-19 in-patients exhibited long duration of viral shedding (LDs, >45 days). We also collected 9 COVID-19 in-patients whose viral shedding durations (SDs) were less than 22 days for comparison. All the patients were identified as laboratory-confirmed and signed an informed consent form at Tongji Hospital, Wuhan, China. And 3 healthy donors who signed an informed consent form as controls. |
| Data exclusions | The sequencing data of patients were processed using Cell Ranger against the GRCh38 human reference genome. Quality of cells were then assessed based on the UMI counts per cell, genes expressed per cell and the proportion of mitochondrial gene counts using Seurat (version 4.0.0). Cells that had UMIs between 500 and 30,000, more than 200 genes expressed and fewer than 15% of UMIs from mitochondrial genes were considered high quality and retained for further analysis. We next identified and removed the doublets following previous described method (Pijuan-Sala et al., 2019). |
| Replication | The samples were distinct by design in this study due to the unique conditions inside the hospitals during COVID-19 outbreak. scRNA-seq profiles of PBMCs samples in each group has at least three samples to draw generalized conclusions. In addition, to better support the generalizability of the observations, we further reclassified, filtered published scRNA-seq data from NCBI GEO database (GSE158055). And to further explore the association between the RP levels and the viral persistence, we integrated bulk-RNA-seq data from 103 independent COVID-19 patients from EMBL-EBI: ERP127339. All attempts at replication were successful. |
| Randomization | Not random. Each sample has served different purposes so no randomization was attempted.<br>1:Cytokine measurements: As of April 30, 2020, a total of 12 non-critical COVID-19 in-patients exhibited long duration of viral shedding (LDs, >45 days). We also collected 38 age- and gender-matched non-critical COVID-19 in-patients whose viral shedding durations (SDs) were less than 21 days for comparison. And 22 healthy donors who signed an informed consent form as controls.<br>2:scRNA-seq: A total of 5 COVID-19 in-patients exhibited long duration of viral shedding (LDs, >45 days). We also collected 9 COVID-19 in-patients whose viral shedding durations (SDs) were less than 22 days for comparison. And 3 healthy donors who signed an informed consent form as controls. |
| Blinding | Investigators were not blinded. Blinding during collection was not needed because conditions were well controlled. Blinding during analysis was not feasible as the differences between samples under different conditions were visually apparent in the trajectories. Blinding is also not necessary because the results are quantitative and did not require subjective judgment or interpretation. |

# Reporting for specific materials, systems and methods

We require information from authors about some types of materials, experimental systems and methods used in many studies. Here, indicate whether each material, system or method listed is relevant to your study. If you are not sure if a list item applies to your research, read the appropriate section before selecting a response.

## Materials & experimental systems

| n/a | Involved in the study |
|---|---|
| x | Antibodies |
| x | Eukaryotic cell lines |
| x | Palaeontology and archaeology |
| x | Animals and other organisms |
|  | x Human research participants |
| x | Clinical data |
| x | Dual use research of concern |

## Methods

| n/a | Involved in the study |
|---|---|
| x | ChIP-seq |
| x | Flow cytometry |
| x | MRI-based neuroimaging |

# Human research participants

Policy information about studies involving human research participants

| | |
|---|---|
| Population characteristics | All COVID-19 patients were divided into two cohorts.<br>1: Cytokine measurements: A total of 12 non-critical COVID-19 in-patients exhibited long duration of viral shedding (LDs, >45 days). We also collected 38 age- and gender-matched non-critical COVID-19 in-patients whose viral shedding durations (SDs) were less than 21 days for comparison.<br>2:scRNA-seq: A total of 5 COVID-19 in-patients exhibited long duration of viral shedding (LDs, >45 days). We also collected 9 COVID-19 in-patients whose viral shedding durations (SDs) were less than 22 days for comparison. |
| Recruitment | Patients were recruited on opportunity for existing pre-established cohorts. Blood samples were obtained in this study. |
| Ethics oversight | This study was reviewed and approved by the Institutional Review Board ofTongji Hospital, Tongji Medical College, Huazhong University of Science and Technology (TJ-IRB20200405) |

Note that full information on the approval of the study protocol must also be provided in the manuscript.
